# Clustering of protein families into functional subtypes using Relative Complexity Measure with reduced amino acid alphabets

**DOI:** 10.1186/1471-2105-11-428

**Published:** 2010-08-18

**Authors:** Aydin Albayrak, Hasan H Otu, Ugur O Sezerman

**Affiliations:** 1 Biological Sciences and Bioengineering, Sabanci University, Orhanli, Tuzla, Istanbul, Turkey; 2 Department of Medicine, BIDMC Genomics Center, Harvard Medical School, Boston, MA 02115, USA; 3 Department of Bioengineering, Istanbul Bilgi University, 34060, Istanbul, Turkey

## Abstract

**Background:**

Phylogenetic analysis can be used to divide a protein family into subfamilies in the absence of experimental information. Most phylogenetic analysis methods utilize multiple alignment of sequences and are based on an evolutionary model. However, multiple alignment is not an automated procedure and requires human intervention to maintain alignment integrity and to produce phylogenies consistent with the functional splits in underlying sequences. To address this problem, we propose to use the alignment-free Relative Complexity Measure (RCM) combined with reduced amino acid alphabets to cluster protein families into functional subtypes purely on sequence criteria. Comparison with an alignment-based approach was also carried out to test the quality of the clustering.

**Results:**

We demonstrate the robustness of RCM with reduced alphabets in clustering of protein sequences into families in a simulated dataset and seven well-characterized protein datasets. On protein datasets, crotonases, mandelate racemases, nucleotidyl cyclases and glycoside hydrolase family 2 were clustered into subfamilies with 100% accuracy whereas acyl transferase domains, haloacid dehalogenases, and vicinal oxygen chelates could be assigned to subfamilies with 97.2%, 96.9% and 92.2% accuracies, respectively.

**Conclusions:**

The overall combination of methods in this paper is useful for clustering protein families into subtypes based on solely protein sequence information. The method is also flexible and computationally fast because it does not require multiple alignment of sequences.

## Background

Proteins that evolve from a common ancestor can change functionality over time [1] and produce highly divergent protein families that can be divided into subfamilies with similar but distinct functions (i.e., functional subfamilies or subtypes) [2]. Identification of subfamilies using protein sequence information can be carried out using phylogenetic methods that can reveal the evolutionary relationship between proteins by clustering similar proteins together in a phylogenetic tree [3-5]. The most common method for identifying similarities in sequences through phylogenetic analysis starts with the construction of a multiple alignment of homologous sequences using a substitution matrix. Multiple alignment scores are then transformed into a distance matrix to construct a phylogenetic tree. Often the branching order of a phylogenetic tree exactly matches the known functional split between proteins [1] and branch lengths are proportional to the extent of evolutionary changes since the last common ancestor.

Multiple sequence alignment (MSA) is constructed using a scoring scheme which reward or penalize each substitution, insertion and deletion to get an optimum alignment of the given sequences. The quality of an MSA is connected to the chosen parameters that are entered manually and an expert handling is almost always required to maintain alignment integrity by observing general trends in each protein family. As such different alignment parameters may yield different phylogenetic trees that are only as good as the MSA that the trees are derived from [6,7].

Phylogenetic analysis is broadly divided into two groups of methods. Algorithms in the first group calculate a matrix representing the distance between each pair of sequences and then transform this matrix into a tree using a tree-clustering algorithm. Algorithms in the first category utilize various distance measures with different models to account for nucleotide or amino acid substitutions. In the second group, the tree that can best explain the observed sequences under the chosen evolutionary model is found by evaluating the fitness of different tree topologies [6,8]. The second category can further be divided into two groups based on the optimality criterion used in tree evaluation: maximum parsimony and maximum likelihood. Under maximum parsimony [9], the preferred phylogenetic tree is the tree that requires the least evolutionary change to explain the observed data whereas under maximum likelihood [9,10], it is the most probable tree under the chosen evolutionary assumption.

The prediction of subfamilies from protein MSAs have been carried out previously by comparing subfamily hidden Markov models, subfamily specific sequence profiles, analyzing positional entropies in an alignment, and ascending hierarchical method [4,5,11,12]. All of these methods require an alignment of biological sequences that assume some sort of an evolutionary model. Computational complexity and the inherent ambiguity of the alignment cost criteria are two major problems in MSA along with controversial evolutionary models that are used to explain them.

A novel approach for phylogenetic analysis based on Relative Complexity Measure (RCM) of whole genomic sequences have been previously proposed by Otu *et al*, that eliminates the need for MSA and produces successful phylogenies on real and simulated datasets [8]. The algorithm employs Lempel-Ziv (LZ) complexity [13] and produces a score for each sequence pair that can be interpreted as the "closeness" of the sequence pairs. Unequal sequence length or different positioning of similar regions along sequences (such as different gene order in genomes) is not an issue as the method has been shown to handle both cases naturally. Moreover, RCM does not use any approximations and assumptions in calculating the distance between sequences. Therefore, RCM utilizes the information contained in sequences and requires no human intervention.

Application of RCM to genomic sequences for phylogenetic analysis was successfully carried out on various datasets containing genomic sequences [8,14]. Moreover, Liu *et al *[15] extended this method further to integrate the hydropathy profile and a different LZ-based distance measure for phylogenetic analysis of protein sequences while Russell *et al *integrated a merged amino acid alphabet containing 11 characters to represent all amino acids to reduce complexity prior to calculating a pairwise distance measure to be used as a pairwise scoring function in determining the order with which sequences should be joined in a multiple sequence alignment problem [16].

Application of RCM to evaluate genomic sequences is relatively straight forward since RCM based on Lempel-Ziv complexity scores can capture each mutation in DNA sequences and register it as an increase in the complexity scores of compared sequences. However, substitution of one residue into another in proteins is tolerable as long as the substituted residue is not highly conserved and physicochemical and structural properties of the substituted and the native residues are not fundamentally different [17-19]. Employment of hydropathy-index-based grouping of residues is one way of a preprocessing requirement to capture only the mutations that would not be tolerated in a protein sequence since LZ algorithm is not capable of accounting for amino acid substitution frequencies and similarity scores. Hence, any application that uses RCM to generate a distance matrix of protein sequences should be linked to treating the sequence with a reduced amino acid alphabet (RAAA) prior to calculating their RCMs.

In this paper, we utilize RCM with different reduced amino acid alphabets and assess RCM's potential in clustering protein families into functional subtypes based solely on sequence data. This method clustered seven well-characterized protein families into their functional subtypes with 92% - 100% accuracy.

## Methods

### Datasets

#### Simulated Dataset

Performance of RCM was tested on a simulated dataset that contains 10 randomly evolved protein sequences from a root sequence of length 500 by using INDELible V1.02 [20]. Simulated protein sequences were generated according to the following parameters:

1. JTT-dcmut [21] was chosen as the amino acid substitution model.

2. Power law insertion/deletion length distribution model with a = 1.7 and maximum allowed insertion/deletion length of 500 were used.

3. Both insertion and deletion rates were set to the default parameter of 0.1 relative to average substitution rate of 1%.

4. Length of the root protein sequence was set to 500.

5. The rooted tree with 10 taxa that reflects the true phylogenetic evolution of the sequences was generated along with the true MSA from which the true tree was inferred.

6. The true MSA was then inputted into ClustalW2 [22] and the bootstrap tree was generated (1000 bootstrap trials, including positions with gaps, and correcting for multiple substitutions)

#### Protein Datasets

RCM was tested on seven protein datasets. Number of sequences, number of subfamilies, average length, standard deviation of sequence lengths and mean percent identities (PID) [23] of sequences for each family are summarized in Table 1. Protein sequences for mandelate racemases, crotonases, haloacid dehalogenases and vicinal oxygen chelates (VOC) were extracted from extensively curated Structure-Function Linkage Database which contains sets of subfamily grouping for a large set of protein families. SFLD contains protein families with a hierarchical classification scheme based on sequence, structure and conserved chemical reactions at the superfamily, subgroup, and family levels [24]. Crotonases and haloacid dehalogenases were filtered such that subfamilies that contain less than 3 sequences or more than 200 sequences were removed to prevent sequence number bias and to reduce computational complexity. Unknown or unspecified amino acids were discarded (21, 22 and 10 occurrences in mandelate racemase, crotonase and VOC family, respectively). The protein sequences for acyl transferase (AT) domains and nucleotidyl cyclases were obtained from reference [25]. The protein sequences in the hard-to-align dataset that contains glycoside hydrolase family 2 (GH2) members were adapted from reference [3]. Expert curated annotations of protein sequences and abbreviations used for sequences in this study are provided in Additional File 1.

**Table 1:** General Properties of the Datasets

Family	# of sequences	# of subfamilies	μ Length	σ Length	μ PID*
Crotonases	467	13	332	87	21
Mandelate racemases	184	8	416	74	27
Vicinal oxygen chelates	309	18	294	108	14
Haloacid dehalogenases	195	14	303	137	12
Nucleotidyl cyclases	75	2	1059	200	21
Acyl transferases	177	2	290	12	41
GH2 hydrolases	33	4	872	160	15

* Mean Percent Identity (μ PID) is the average of all pairwise sequence identities in a given family.

### Reduced Amino Acid Alphabets

Sequence space of proteins is redundant and generates only a limited number of folds, domains, and structures [26]. Various strategies have been devised that take a coarse-grained approach to account for the degeneracy of sequences by grouping similar amino acids together [17-19,27-30]. Grouping is usually carried out based on structural and physiochemical similarities of amino acids [28]. Grouping of amino acids in sequence space can help develop prediction methods for various sequence determinants and decrease the amount of search space in procedures employed in directed evolution experiments [26,31]. One of the finest examples is the reduction of amino acid alphabet into a binary code that is composed of characters representing polar and non-polar amino acid residues [27]. Grouping of amino acid residues has also been used extensively in Hydrophobic-Polar (HP) lattice model to explain the hydrophobic collapse theory of protein folding [32].

A recent study was carried out by Peterson *et al *to test the performance of over 150 RAAAs on the sequence library from DALIpdb90 database and showed that RAAAs improves sensitivity and specificity in fold prediction between protein sequence pairs with high structural similarity and low sequence identity [33].

We tested performances of six amino acid reduction schemes with 15 different level of groupings to separate proteins into functional subfamilies (Table 2). These included three top performing RAAA (HSDM17, SDM12, GBMR4) from reference [33] and three random RAAA of size 4.

**Table 2:** Reduced Amino Acid Alphabets

Scheme	Size	Matrix	Gaps^#^	Reference
ML*	4,8,10,15	BL50	12/2	[28]
EB^§^	13,11,9,8,5	BL62	11/1	[18]
HSDM*	17	HSDM	19/1	[29]
SDM*	12	SDM	7/1	[29]
GBMR*	4	BL62	11/1	[30]
RANDOM^§^	4,4,4	BL62	11/1	This study

Reduced amino acid schemes used in this study.* Substitution matrices for these reduced alphabets were obtained from reference [33]. ^§ ^BL62 frequency counts were used to derive these substitution matrices using the formula outlined in reference [33]. ^#^Gap opening/gap extension penalties used for MSAs in ClustalW2.

### Substitution Matrices

Amino acids that are within the same group in a RAAA are considered identical [33]. Substitution matrices that assign the same similarity score to each amino acid within the same group were obtained from reference [33]. For those RAAAs in the EB scheme and the three random RAAAs, new substitution matrices were created from BLOSUM62 frequency counts using the same procedure outlined in reference [33].

### Lempel-Ziv Complexity

In this paper, a normalized distance measure that was previously used for phylogenetic tree construction of whole genome sequences was employed. The distance measure was based on Lempel-Ziv [34] complexity and was known to accurately cluster all related genomic sequences under one branch of the tree [8].

Lempel-Ziv (LZ) complexity score of a sequence is obtained by counting the number of steps required to generate a copy of the primary sequence starting from a null state. At each step, an amino acid or a series of amino acids are copied from the subsequence that has been constructed thus far allowing for a single letter innovation. The number of steps needed to obtain the whole sequence is identified as the LZ-complexity score of the given sequence. The exhaustive library of a sequence is defined as the smallest number of distinct amino acid or amino acid combinations required to construct the sequence using a copying process described by Lempel and Ziv [34]. For example, the LZ-complexity of the simple sequence 'AAILNAIIANNL' would be obtained as shown in Table 3. Since seven steps are needed to generate the whole sequence, the LZ-complexity score for this sequence is 7. The LZ-complexity of a sequence 'X' compared to a sequence 'Y' is known as the RCM of 'X' with respect to 'Y'. This is the number of steps required to construct sequence 'X' beginning with 'Y' instead of a null sequence. Five different distance metrics have been suggested by Otu *et al *[8], however, this work used only the following normalized distance metric that accounts for the differences in sequence lengths:

**Table 3:** Lempel-Ziv Complexity

Sequence X = AAILNAIIANNL	
**Exhaustive History**	**Complexity**
A	1
AI	2
L	3
N	4
AII	5
AN	6
NL	7

**H_E_(X)**	**C(X) = 7**

The exhaustive library construction and Lempel-Ziv complexity score calculation of sequence X.

$$D_{XY}=\frac{c(XY)+c(YX)-c(X)-c(Y)}{\frac{c(XY)+c(YX)}{2}}$$

where c(XY) and c(YX) are RCM of X appended to Y and Y appended to X, respectively. Remaining four LZ-based distance measures defined in Out *et al *performed slightly worse than the above distance (data not shown). Although in performance between five measures were not significant, we adopted the aforementioned distance for its ability to account for length variance.

### Distance Matrix & Phylogenetic Tree

The relative complexity measure (RCM) for creation of the distance matrix was utilized as previously described [8]. Phylogenetic trees were generated from distance matrices using neighbor-joining [35] program of the phylogeny inference package, PHYLIP 3.68 [36]. Un-rooted trees were rooted with midpoint rooting by placing the root halfway between the two most distinct taxa. Midpoint-rooted trees were converted to cladograms (i.e., branch lengths are discarded) using the Retree program of PHYLIP package [36]. Phylogenetic trees for all protein families and RAAAs are shown in supplementary materials (Additional File 2) in Newick format and can be visualized with a tree-drawing program.

### ClustalW2

Protein sequences in each family were aligned using ClustalW2 [22] for comparison with RCM. MSAs were performed using updated substitution matrices with gap extension and gap opening penalties provided in Table 2. Bootstrap analyses were carried out 100 times and trees containing bootstrap values were created using ClustalW2 with the neighbor-joining clustering algorithm. For convenience, MSAs that were carried out using ClustalW2 will be referred as the MSA or the MSA method for the rest of the article.

### Tree Based Classification (TBC)

TBC algorithm [4] was used to check the accuracy of each tree in separating protein families into subfamilies. TBC divides a tree into disjoint subtrees and assigns a protein subfamily to a subtree that maximizes the number of true positives when the proportions of *fp*/(*tp*+*fp*) and *fn*/(*tp*+*fn*) are both equal to 0.5 for a given subtree, where *fp *is the number of false positives, *fn *is the number of false negatives and *tp *is the number of true positives. Above proportions correspond to the "maximal allowed contamination" level that minimizes the TBC error over the whole tree.

TBC requires a bifurcating tree of sequences in a protein family and an attribute file that contains expert curated assignment of each sequence to a particular subfamily. TBC accuracy (i.e., the percentage of correctly classified sequences) is the primary performance measure to evaluate the division of protein families into subtypes using the TBC algorithm. TBC accuracy is equal to 1- %TBC error where %TBC error is the total number of *fp*, *fn*, and *unclassified sequences *divided by the total number of sequences. For a detailed analysis of the TBC algorithm, refer to reference [4].

### Protocol

The proposed algorithm operates on a set of sequences in FASTA format. After one of the alphabets given in Table 1 is applied to all the sequences in the dataset, RCMs are calculated and used to obtain the distance between each pair for the neighbor-joining clustering to create a phylogenetic tree. For each RAAA, a single tree based on RCM is generated and analyzed using TBC algorithm to determine how well it clusters different subfamilies under different branches of the tree.

For simulated dataset, three phylogenetic trees were compared: The true tree generated by INDELible, the bootstrap tree and the RCM tree. INDELible creates a true MSA of the simulated protein sequences. This alignment was used in ClustalW2 and bootstrapped 1000 times and the resulting tree was called the bootstrap tree. The third tree is the RCM tree that was generated by the proposed approach.

For seven protein datasets, first, the original fasta sequences were used to calculate RCMs and their associated RCM trees. Second, the original fasta sequences were re-coded using different RAAAs (Table 2) and the reduced sequences were used to calculate their RCMs and the associated RCM trees.

A similar procedure was applied to the phylogenetic trees using the MSA method. For each protein family, MSA was carried out using the corresponding substitution matrices and gap penalties provided in Table 2. MSA-based trees were created following bootstrap analysis (100 replicates) with ClustalW2.

**Figure 1:**
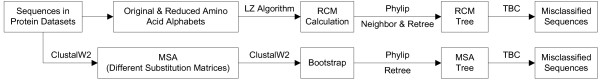
**Protocol Overview**. For RCM, the original sequences and sequences recoded with reduced alphabets are used to calculate RCM-based distances which are then inputted sequentially to the Neighbor-Joining and Retree programs of the PHYLIP v3.68 package. For MSA, first, alignments are carried out using ClustalW2 with substitution matrices corresponding to each amino acid alphabet. Following bootstrap analysis with ClustalW2, Retree program is used to root the trees with midpoint rooting and to discard branch lengths. Each phylogenetic tree is then inputted to the TBC algorithm along with its attribute file that shows the expert assignment of each sequence to each family to calculate the TBC error.

Finally, for each family, a total of 16 phylogenetic trees (1 for 20-letter alphabet, 12 for RAAAs, and 3 for random RAAAs) for each method are generated and checked how well they separated families into subfamilies. A summary of the overall workflow is depicted in Figure 1.

## Results and Discussion

### Simulated Dataset

**Figure 2:**
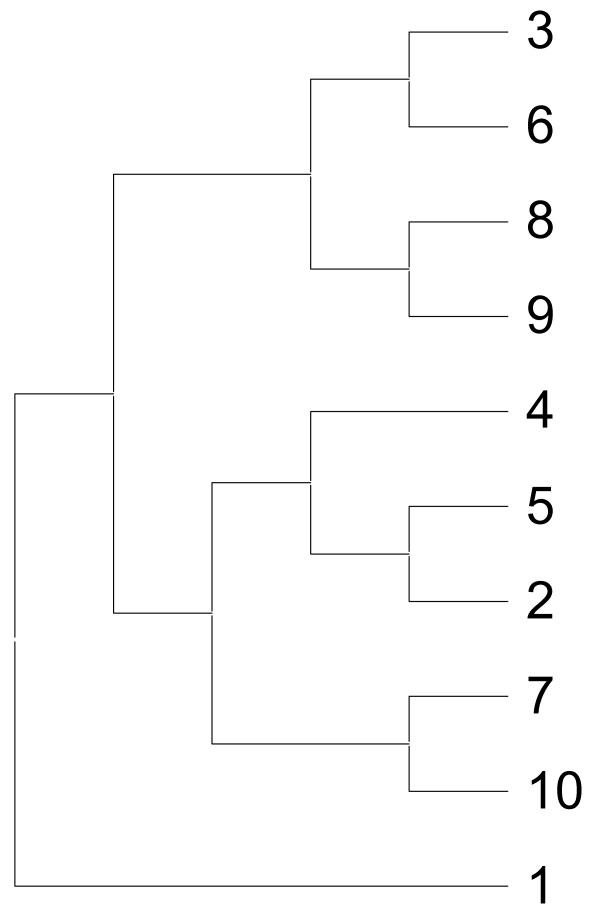
**Tree topology of the simulated dataset**. The identical topology of the three phylogenetic trees (i.e., RCM tree, bootstrap tree and true tree) for the simulated dataset is shown.

Phylogenetic analysis of protein sequences has been intimately connected with MSA. A phylogenetic tree is generated from an evolutionary distance matrix using MSA of sequences. However, for real biological datasets, the true tree is rarely known. Therefore, protein sequence evolution was simulated to study the reliability of the RCM method. A simulated protein dataset containing 10 protein sequences was generated to show that RCM coupled with a RAAA can produce a phylogenetic tree (RCM tree) that is consistent with the true tree and the bootstrap tree. The true tree is produced by INDELible and is the original tree that reflects the evolution of 10 simulated sequences. On the other hand, the bootstrap tree is the tree that was produced by ClustalW2 using the true MSA implied by INDELible. The bootstrap tree is identical to the true tree and the bootstrap supports for all branches are high reflecting the consistency [37] in the branching. The RCM tree was produced by the alignment-free RCM approach. The RCM tree is identical to both the true tree and the bootstrap tree reflecting its potential for use in phylogenetic analysis of protein sequences. The tree topology of only one of the trees is shown in Figure 2 since they are all identical.

### Performance of the RCM approach

**Figure 3:**
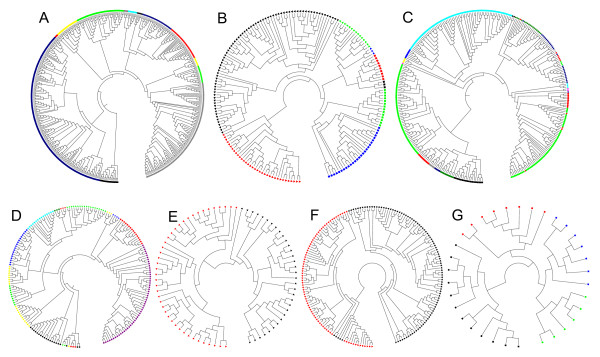
**Phylogenetic trees of protein families**. RCM trees were drawn using ML15 alphabet. For each family, the taxa corresponding to different subfamilies are colored differently. (A) Crotonases (B) Mandelate racemases (C) Vicinal oxygen chelates (D) Haloacid dehalogenase (E) Nucleotidyl cyclases (F) Acyl transferases (G) GH2 hydrolases

We applied the RCM approach to seven protein datasets. RCM method showed an efficient division of protein families into subfamilies using RAAAs. Phylogenetic trees of the seven protein families using RCM approach are shown in Figure 3 for ML15 alphabet. Detailed comparison of RCM with MSA in terms of TBC accuracy, the number and percentage of TBC error for each RAAA and each dataset is provided in Additional File 3.

#### Crotonases

Members of crotonase family contain 467 protein sequences from 13 different subfamilies and catalyze diverse metabolic reactions with certain family members displaying dehalogenase, hydratase, and isomerase activities. TBC accuracy varied between 96.4% and 100% for RCM. The top performing RAAA with the smallest size was GBMR4 that resulted in 100% TBC accuracy. TBC accuracy was 100% for all RAAAs tested with MSA.

#### Mandelate Racemases

The mandelate racemase dataset contains 184 sequences that are assigned to 8 expert curated subfamilies. All mandelate racemases contain a conserved histidine, presumably acting as an active site base [38]. When the RCM approach was tested on mandelate racemases, all resulting trees showed correct assignment of functional subfamilies into 8 different clusters with 100% accuracy using all alphabets except GBMR4 that resulted in 96.7% TBC accuracy.

#### Vicinal oxygen chelates (VOC)

VOC family contains 309 sequences from 18 different subfamilies. The number of TBC accuracy varied between 77.7% and 92% for RCM and 81.9% to 91.3% for MSA. Members of VOC have an average sequence length of 294 amino acids and a mean PID of 14% (Table 1). The low PID and the highly divergent nature of this family make its subfamilies susceptible to misclassification more than other families based on sequence information alone. In this dataset, EB8 performed better than 20-letter alphabet (92.2% vs. 91.3%) with RCM while GBMR4, ML4, EB8, EB, EB13 and 20-letter alphabets resulted in 91.3% TBC errors with MSA.

#### Haloacid dehalogenases

Haloacid dehalogenases contains 195 sequences that belong to 14 different subfamilies. Haloacid dehalogenase family is similar to VOCs in its highly divergent nature based on the low mean PID (12%) that places the sequences in this family in the "twilight zone" to infer any relation between sequences based on sequence information alone. ML15 was the best performing RAAA for RCM with 96.9% accuracy (Table 4). The size of the best performing RAAA for this family is larger compared to other families hinting that highly divergent sequences may require larger alphabets with lower level of grouping.

**Table 4:** TBC errors for top performing RAAA

		Crotonases		Mandelate racemases		Vicinal oxygen chelates		Haloacid dehalogenases		Nucleotidyl cyclases		Acyl transferases		GH2 hydrolases	
		
		RCM	MSA	RCM	MSA	RCM	MSA	RCM	MSA	RCM	MSA	RCM	MSA	RCM	MSA
20 letter	Accuracy	100	100	100	100	91.6	91.3	93.3	99.5	100	100	91.5	97.2	87.9	100
	Error	0	0	0	0	8.4	8.7	6.7	0.5	0	0	8.5	2.8	12.1	0

Statistics for top performing RAAA	Accuracy	100	100	100	100	**92.2**	91.3	96.9	**99.5**	100	100	97.2	97.2	100	100
	Error	0	0	0	0	7.8	8.7	3.1	0.5	0	0	2.8	2.8	0	0

Top performing RAAAs	RAAA	GBMR4	ML4 GBMR4	ML4	GBMR4 ML4	EB8	GBMR4 ML4	ML15	ML8	ML4 GBMR4	GBMR4	ML4	ML4 GBMR4	ML4 GBMR4	ML4 GBMR4

TBC accuracy and percentage of TBC error are reported for the 20-letter alphabet and the top performing RAAA. If two RAAAs with the same size have identical TBC accuracies, both RAAAs are reported at the final row in the table. Bold entries correspond to top performers using RCM and MSA for the specified datasets

#### Nucleotidyl cyclases

Nucleotidyl cyclase family has two functional subfamilies, adenylate and guanylate cyclases that correspond to use of the substrates ATP and GTP respectively. The nucleotidyl cyclase family with 33 adenylate cyclases and 42 guanylate cyclases was clustered into two distinct subfamilies with 100% accuracy using both methods and all RAAAs except EB5 and EB8 for RCM and ML4 and EB5 for MSA, all of which resulted in 98.7% accuracy (Table 4). Moreover, the clustering result for the nucleotidyl cyclases are in agreement with the result obtained previously by the MSA-dependent clustering algorithm that uses the residues with the highest evolutionary split statistic to split protein families into functional subfamilies [25].

#### Acyl transferases (AT)

The AT domains of Type I modular polyketide synthases are responsible for the substrate selection. Most incorporate either a C2 unit (malonyl-CoA substrate) or a C3 unit (methylmalonyl-CoA substrate). The choice of substrate can be deduced from the chemical structure of the polyketide product [25]. In the acyl transferase dataset, 99 of the 177 sequences use C2 units whereas 78 use C3 units as substrate.

Previously, Goldstein *et al *[25] used evolutionary split statistic and clustered the AT domains into 2 subfamilies with 2 false assignments for the 5 residue-long motif. The number of false assignments increased to 5 with increasing motif length (up to 30-residue long) suggesting that the utilization of a larger motif increases the noise and error rate. As such, inclusion of only 5 residues (less noise) with high split statistics increases the assignment accuracy (5 vs. 2 false assignments).

A similar trend is observed in the case of RCM. While the TBC accuracy for AT domains was only 91% (15 false assignments) with the 20-letter alphabet (Table 4), the accuracy increased to 97% (5 false assignments) with the utilization of the ML4, ML8, EB9, ML10, EB11, SDM12, EB13, and HSDM17 alphabets. Furthermore, 4 of the 5 misclassified sequences using the above reduced alphabets are contained in the 2, 3 and 4 false assignments produced by the Goldstein *et al *'s approach using the 5,10 and 15 residue-long motifs, respectively. Although the accuracy was higher previously, it should be noted that the RCM approach did neither require an MSA of sequences nor any other sequence-based statistics. The accuracy was 97.2% for MSA using the top performing RAAAs. There was no immediate evidence suggesting a specific characteristic for incorrectly classified sequences.

#### Glycoside hydrolase family 2 (GH2)

The final dataset contains 33 members of the GH2 family with a (β/α)_8 _fold. The subfamilies and the number of sequences from each subfamily are β-galactosidases (6), β-mannosidases (12), β-glucuronidases (7) and exo-β-D-glucosaminidases (8). This dataset was used previously and chosen because it was cited as a "hard-to-align" dataset by classical alignment approaches [3]. The GH2 family was clustered into 4 functional subfamilies with 100% accuracy using ML4 and GBMR4 - the two top performing RAAAs - with RCM (Table 4). TBC accuracy was 100% for all RAAAs tested with MSA.

### The effect of the size of the RAAA on clustering performance

The comparison of RCM with MSA in terms of TBC accuracy and the percentage of TBC error are summarized in Table 4 for the 20-letter alphabet and the top performing RAAA with the minimum size. In cases where two RAAAs of the same size give identical TBC results, both of them are reported. Three trends can be observed from the data in Table 4.

First, for five of the seven families (crotonases, mandelate racemases, nucleotidyl cyclases, acyl transferases, and GH2 hydrolases), both methods perform equally well comparably. For VOC, RCM outperforms MSA while for haloacid dehalogenases, MSA slightly outperforms RCM. It is important to note that both VOCs and dehalogenases have the two lowest mean PIDs (12% vs. 14%) and low mean sequence lengths with large standard deviation. Low PID and low sequence length are two features in alignments that render inference of relationship based only on sequence information difficult. Nonetheless, TBC accuracies of both families with their respective top performing RAAAs are comparable to the results obtained from the protein families with higher mean PIDs and longer mean sequence lengths.

Second, either ML4 or GBMR4 is sufficient to obtain high TBC accuracy for all datasets except VOCs and haloacid dehalogenases. Indeed, apart from the aforementioned families, ML4 and GBMR4 can produce either identical or better results than all other alphabets using either RCM or MSA, implying that as little as an alphabet size of 4 would be sufficient to capture most of the sequence information that might yield considerable improvements in inferring relationship based on sequence information when both mean PID and the length of the aligned regions in an MSA is above a certain threshold.

Third, for the datasets with low mean PIDs and average sequence lengths, a larger RAAA size may be required to obtain identical or better results than the 20-letter alphabet using both RCM and MSA. This is especially evident with the RCM approach. While the minimum RAAA size of the top performer was 4 for 5 datasets that have relatively higher average sequence lengths and mean PIDs, it increases to 8 (EB8) for VOCs and 15 (ML15) for haloacid dehalogenases that have mean PIDs of 14% and 12%, respectively. Moreover, a subtle but a similar trend is also evident in the case of MSA. While the alphabet size of the top performer was 4 (GBMR4, ML4) for VOCs, it increased to 8 (ML8) for haloacid dehalogenases, implying that a larger RAAA size may perform better on sequences with lower sequence identities.

It is also interesting to note that the average TBC error for mandelate racemases, nucleotidyl cyclases and hydrolases with three random alphabets of size 4 varied between 0% and 15.6% for the MSA method. While the groupings of amino acids in the random alphabets do not have any physicochemical or structural significance that can justify this overall performance, the low percent TBC error may suggest that some subfamilies of these protein families may be very tight with small distances between their sequences while larger distance between different subfamilies. This scenario coupled with the relatively longer sequences (top three families in terms of mean sequence length) within these families may generate sufficiently long aligned regions with enough informative sites that can result in a tree that correctly assigns subfamilies even the reduced alphabet groupings do not have any structural or biological meaning.

However, the trend of low TBC error is not apparent using RCM with random alphabets. TBC errors of different protein families using random RAAAs (average of three random alphabets) were significantly higher than TBC errors using biologically meaningful reduced alphabets for all the families except racemases and nucleotidyl cyclases, both of which overlap with the results obtained with MSA.

Performance of RCM approach with different RAAAs to cluster protein families into functional subfamilies is eminent. Yet, it must be noted that there is no uniformly superior algorithm for tree-based subfamily clustering and that simple protein similarity measures combined with hierarchical clustering produce trees with reasonable and often high accuracy [4]. Furthermore, if much time has passed since the evolution of different subfamilies, then sequences may have diverged beyond the point where simple phylogenetic analysis cannot easily give a clear distinction of subfamilies.

## Conclusions

The application of RCM in generating meaningful phylogenetic trees has been previously tested on genomic sequences and made RCM a good alternative to MSA-based phylogenetic analysis. However, integration of RCM to measure the closeness of protein sequences was simply problematic due to the lack and difficulty of accounting for amino acid substitutions. In this paper, we introduced an RAAA-based approach as a preprocessing of protein sequences prior to calculating pairwise RCMs. Utilization of an RAAA that is consistent with the structure and function of the proteins or an RAAA that reflects the general trends in specific protein families under study can result in successful phylogenies that can cluster each protein superfamily into functional subfamilies.

In finding functional subtypes of a protein family, it is often of interest to find out if the mechanisms that manipulate a certain clustering are of evolutionary or functional origin. Although these two signals may be overlapping and hard to separate, RCM could be used to address this issue by finding differences in exhaustive histories in two sequences when they are concatenated. The "words" that result in an observed difference can then be analyzed and correlated to a functional and/or evolutionary origin. We believe future work can focus in this direction building on the current approach that does not attempt to trace back the origin of differentiating sequence signals but provides a powerful clustering method of protein families into functional subtypes without using multiple sequence alignment.

## Authors' contributions

UOS and HHO participated in the design of the study and supervised all the experiments. AA performed all the experiments and wrote the initial manuscript and the final manuscript. HHO provided the LZ algorithm, revised the first and the final manuscript. All authors read and approved the final manuscript.

## Supplementary Material

Additional file 1 **Fasta header mapping**. This file contains the fasta header abbreviations for protein families and expert assignment of sequences to each subfamily. Some bioinformatics programs that take fasta files as input have fasta header size limitations ranging from 8 to 10 characters long. Click here for file

Additional file 2 **Phylogenetic trees for all the datasets in Newick**. Phylogenetic tree files for all families are presented in Newick format. For simulated dataset, there are 3 phylogenetic trees. For each protein dataset, there are 32 phylogenetic trees: 16 RCM trees and 16 MSA trees. All trees reflect only the tree topology (i.e., Branch lengths are discarded). Click here for file

Additional file 3 **TBC errors for all families and all RAAAs**. Detailed comparison of RCM and MSA is reported in terms of the number and percentage of TBC error for every protein family and RAAA under consideration. Click here for file

## References

[B1] Wallace IM Higgins DG Supervised multivariate analysis of sequence groups to identify specificity determining residues BMC Bioinformatics 2007 8 135 10.1186/1471-2105-8-135 17451607 PMC1878507

[B2] Georgi B Schultz J Schliep A Partially-supervised protein subclass discovery with simultaneous annotation of functional residues BMC Struct Biol 2009 9 68 10.1186/1472-6807-9-68 19857261 PMC2777906

[B3] Kelil A Wang S Brzezinski R Fleury A CLUSS: clustering of protein sequences based on a new similarity measure BMC Bioinformatics 2007 8 286 10.1186/1471-2105-8-286 17683581 PMC1976428

[B4] Lazareva-Ulitsky B Diemer K Thomas PD On the quality of tree-based protein classification Bioinformatics 2005 21 9 1876 1890 10.1093/bioinformatics/bti244 15647305

[B5] Wicker N Perrin GR Thierry JC Poch O Secator: a program for inferring protein subfamilies from phylogenetic trees Mol Biol Evol 2001 18 8 1435 1441 11470834 10.1093/oxfordjournals.molbev.a003929

[B6] Brocchieri L Phylogenetic inferences from molecular sequences: review and critique Theor Popul Biol 2001 59 1 27 40 10.1006/tpbi.2000.1485 11243926

[B7] Baldauf SL Phylogeny for the faint of heart: a tutorial Trends Genet 2003 19 6 345 351 10.1016/S0168-9525(03)00112-4 12801728

[B8] Otu HH Sayood K A new sequence distance measure for phylogenetic tree construction Bioinformatics 2003 19 16 2122 2130 10.1093/bioinformatics/btg295 14594718

[B9] Felsenstein J Evolutionary trees from DNA sequences: a maximum likelihood approach J Mol Evol 1981 17 6 368 376 10.1007/BF01734359 7288891

[B10] Nei M Phylogenetic analysis in molecular evolutionary genetics Annu Rev Genet 1996 30 371 403 10.1146/annurev.genet.30.1.371 8982459

[B11] Hannenhalli SS Russell RB Analysis and prediction of functional sub-types from protein sequence alignments J Mol Biol 2000 303 1 61 76 10.1006/jmbi.2000.4036 11021970

[B12] Brown DP Krishnamurthy N Sjolander K Automated protein subfamily identification and classification PLoS Comput Biol 2007 3 8 e160 10.1371/journal.pcbi.0030160 17708678 PMC1950344

[B13] Ziv J Lempel A A universal algorithm for sequential data compression IEEE Trans Inf Theory 1977 23 337 343 10.1109/TIT.1977.1055714

[B14] Bastola DR Otu HH Doukas SE Sayood K Hinrichs SH Iwen PC Utilization of the relative complexity measure to construct a phylogenetic tree for fungi Mycol Res 2004 108 Pt 2 117 125 10.1017/S0953756203009079 15119348

[B15] Liu N Wang T Protein-based phylogenetic analysis by using hydropathy profile of amino acids FEBS Lett 2006 580 22 5321 5327 10.1016/j.febslet.2006.08.086 16979630

[B16] Russell DJ Otu HH Sayood K Grammar-based distance in progressive multiple sequence alignment BMC Bioinformatics 2008 9 306 10.1186/1471-2105-9-306 18616828 PMC2478692

[B17] Wang J Wang W A computational approach to simplifying the protein folding alphabet Nat Struct Biol 1999 6 11 1033 1038 10.1038/14918 10542095

[B18] Etchebest C Benros C Bornot A Camproux AC de Brevern AG A reduced amino acid alphabet for understanding and designing protein adaptation to mutation Eur Biophys J 2007 36 8 1059 1069 10.1007/s00249-007-0188-5 17565494

[B19] Li T Fan K Wang J Wang W Reduction of protein sequence complexity by residue grouping Protein Eng 2003 16 5 323 330 10.1093/protein/gzg044 12826723

[B20] Fletcher W Yang Z INDELible: a flexible simulator of biological sequence evolution Mol Biol Evol 2009 26 8 1879 1888 10.1093/molbev/msp098 19423664 PMC2712615

[B21] Kosiol C Goldman N Different versions of the Dayhoff rate matrix Mol Biol Evol 2005 22 2 193 199 10.1093/molbev/msi005 15483331

[B22] Larkin MA Blackshields G Brown NP Chenna R McGettigan PA McWilliam H Valentin F Wallace IM Wilm A Lopez R Clustal W and Clustal X version 2.0 Bioinformatics 2007 23 21 2947 2948 10.1093/bioinformatics/btm404 17846036

[B23] Eddy SR Profile hidden Markov models Bioinformatics 1998 14 9 755 763 10.1093/bioinformatics/14.9.755 9918945

[B24] Pegg SC Brown SD Ojha S Seffernick J Meng EC Morris JH Chang PJ Huang CC Ferrin TE Babbitt PC Leveraging enzyme structure-function relationships for functional inference and experimental design: the structure-function linkage database Biochemistry (Mosc) 2006 45 8 2545 2555 10.1021/bi052101l 16489747

[B25] Goldstein P Zucko J Vujaklija D Krisko A Hranueli D Long PF Etchebest C Basrak B Cullum J Clustering of protein domains for functional and evolutionary studies BMC Bioinformatics 2009 10 335 10.1186/1471-2105-10-335 19832975 PMC2770074

[B26] Strelets VB Shindyalov IN Lim HA Analysis of peptides from known proteins: clusterization in sequence space J Mol Evol 1994 39 6 625 630 10.1007/BF00160408 7807551

[B27] Dill KA Theory for the folding and stability of globular proteins Biochemistry (Mosc) 1985 24 6 1501 1509 10.1021/bi00327a032 3986190

[B28] Murphy LR Wallqvist A Levy RM Simplified amino acid alphabets for protein fold recognition and implications for folding Protein Eng 2000 13 3 149 152 10.1093/protein/13.3.149 10775656

[B29] Prlic A Domingues FS Sippl MJ Structure-derived substitution matrices for alignment of distantly related sequences Protein Eng 2000 13 8 545 550 10.1093/protein/13.8.545 10964983

[B30] Solis AD Rackovsky S Optimized representations and maximal information in proteins Proteins 2000 38 2 149 164 10.1002/(SICI)1097-0134(20000201)38:2<149::AID-PROT4>3.0.CO;2-# 10656262

[B31] Munoz E Deem MW Amino acid alphabet size in protein evolution experiments: better to search a small library thoroughly or a large library sparsely? Protein Eng Des Sel 2008 21 5 311 317 10.1093/protein/gzn007 18375453 PMC4478448

[B32] Lau KF Dill KA A lattice statistical mechanics model of the conformational and sequence spaces of proteins Macromolecules 1989 22 10 3986 3997 10.1021/ma00200a030

[B33] Peterson EL Kondev J Theriot JA Phillips R Reduced amino acid alphabets exhibit an improved sensitivity and selectivity in fold assignment Bioinformatics 2009 25 11 1356 1362 10.1093/bioinformatics/btp164 19351620 PMC2732308

[B34] Lempel A Ziv J On the Complexity of Finite Sequences IEEE Trans Inf Theory 1976 22 1 75 81 10.1109/TIT.1976.1055501

[B35] Saitou N Nei M The neighbor-joining method: a new method for reconstructing phylogenetic trees Mol Biol Evol 1987 4 4 406 425 3447015 10.1093/oxfordjournals.molbev.a040454

[B36] Felsenstein J PHYLIP - Phylogeny Inference Package (Version 3.2) Cladistics 1989 5 164 166

[B37] Holmes S Bootstrapping Phylogenetic Trees: Theory and Methods Stat Sci 2003 18 2 241 255 10.1214/ss/1063994979

[B38] Gerlt JA Babbitt PC Divergent evolution of enzymatic function: mechanistically diverse superfamilies and functionally distinct suprafamilies Annu Rev Biochem 2001 70 209 246 10.1146/annurev.biochem.70.1.209 11395407

